# Correction: Saksida et al. Is Pupil Response to Speech and Music in Toddlers with Cochlear Implants Asymmetric? *Audiol. Res.* 2025, *15*, 108

**DOI:** 10.3390/audiolres15050134

**Published:** 2025-10-10

**Authors:** Amanda Saksida, Marta Fantoni, Sara Ghiselli, Eva Orzan

**Affiliations:** 1 Institute for Maternal and Child Health—IRCCS “Burlo Garofolo”–Trieste, via dell’Istria 65/1, 34137 Trieste, Italy; 2 AUSL Piacenza—Guglielmo da Saliceto Hospital, Via Antonio Anguissola 15, 29121 Piacenza, Italy

**Extra Affiliation**

In the published publication [1], there was an error regarding the affiliation of Amanda Saksida; the updated affiliation should remove “2 Educational Research Institute, Gerbičeva 62, 1000 Ljubljana, Slovenia”. The authors state that the scientific conclusions are unaffected. This correction was approved by the Academic Editor. The original publication has also been updated.
