# Supplementary material for: Is Pupil Response to Speech and Music in Toddlers with Cochlear Implants Asymmetric?
Source: Audiol Res. 2025 Aug 14;15(4):108. doi: 10.3390/audiolres15040108 (PMC12382946; doi:10.3390/audiolres15040108)
Supplement: Supplementary file 1 [file audiolres-15-00108-s001.zip › audiolres-3688563-supplementary.pdf]

Supplementary material S1

Model M1

```
Linear mixed model fit by REML. t-tests use Satterthwaite's method ['lmerModLmerTest']
Formula: pupil ~ session * signal * background + (1 | subj)
   Data: allmeans[allmeans$signal != "silence", ]

REML criterion at convergence: 20.8

Scaled residuals: 
     Min       1Q   Median       3Q      Max 
-2.76506 -0.65440 -0.02322  0.62536  2.61712 

Random effects:
 Groups   Name        Variance Std.Dev.
 subj     (Intercept) 0.008955 0.09463 
 Residual             0.053830 0.23201 
Number of obs: 227, groups:  subj, 9

Fixed effects:
                                        Estimate Std. Error        df t value
(Intercept)                              0.30792    0.06829  86.47962   4.509
sessionleft ear                          0.13560    0.08050 212.07830   1.684
signalmusic                              0.03968    0.07883 212.20585   0.503
backgroundnoise                         -0.01557    0.07144 212.35655  -0.218
sessionleft ear:signalmusic             -0.16864    0.10761 211.41412  -1.567
sessionleft ear:backgroundnoise         -0.07591    0.09608 211.59599  -0.790
signalmusic:backgroundnoise              0.01498    0.09672 212.41651   0.155
sessionleft ear:signalmusic:backgroundnoise  0.06843    0.13183 211.63590   0.519
                                        Pr(>|t|)    
(Intercept)                             2.03e-05 ***
sessionleft ear                           0.0936 .  
signalmusic                               0.6152    
backgroundnoise                           0.8277    
sessionleft ear:signalmusic               0.1186    
sessionleft ear:backgroundnoise           0.4304    
signalmusic:backgroundnoise               0.8770    
sessionleft ear:signalmusic:backgroundnoise   0.6043    
---
Signif. codes:  0 ‘***’ 0.001 ‘**’ 0.01 ‘*’ 0.05 ‘.’ 0.1 ‘ ’ 1

Correlation of Fixed Effects:
            (Intr) sssnle sgnlms bckgrn sssnlfter:s sssnlfter:b sgnlm:
sessinlfter -0.663                                                    
signalmusic -0.678  0.573                                             
backgrondns -0.748  0.633  0.647                                      
sssnlfter:s  0.491 -0.745 -0.728 -0.470                               
sssnlfter:b  0.553 -0.836 -0.478 -0.740  0.624                        
sgnlmsc:bck  0.556 -0.470 -0.817 -0.742  0.594       0.548            
ser:sgnlms: -0.405  0.610  0.597  0.542 -0.817      -0.730      -0.731
```

```
Analysis of Deviance Table (Type II Wald chisquare tests)

Response: pupil
                           Chisq Df Pr(>Chisq)
session                   0.4854  1    0.48597
signal                    0.2645  1    0.60703
background                0.7912  1    0.37373
session:signal            3.9292  1    0.04745 *
session:background        0.3618  1    0.54750
signal:background         0.6128  1    0.43372
session:signal:background 0.2694  1    0.60373
---
Signif. codes:  0 ‘***’ 0.001 ‘**’ 0.01 ‘*’ 0.05 ‘.’ 0.1 ‘ ’ 1
```

Model M1a

```
Linear mixed model fit by REML. t-tests use Satterthwaite's method ['lmerModLmerTest']
Formula: pupil ~ session + (1 | subj)
   Data: allmeans[allmeans$signal == "speech", ]

REML criterion at convergence: -6.7

Scaled residuals:
     Min       1Q   Median       3Q      Max
-2.52090 -0.67261 -0.02651  0.66297  2.83454

Random effects:
 Groups   Name        Variance Std.Dev.
 subj     (Intercept) 0.01039  0.1019
 Residual             0.04659  0.2159
Number of obs: 112, groups:  subj, 9

Fixed effects:
               Estimate Std. Error        df t value Pr(>|t|)
(Intercept)     0.30528    0.04568  14.59974   6.682  8.4e-06 ***
sessionleft ear 0.08190    0.04117 103.67100   1.989   0.0493 *
---
Signif. codes:  0 ‘***’ 0.001 ‘**’ 0.01 ‘*’ 0.05 ‘.’ 0.1 ‘ ’ 1

Correlation of Fixed Effects:
            (Intr)
sessinlfter -0.487
Analysis of Deviance Table (Type II Wald chisquare tests)

Response: pupil
         Chisq Df Pr(>Chisq)
session 3.9567  1    0.04669 *
---
Signif. codes:  0 ‘***’ 0.001 ‘**’ 0.01 ‘*’ 0.05 ‘.’ 0.1 ‘ ’ 1
```

Model M2

```
Call:
lm(formula = pupil ~ (age + CIthreshold + Ci1time + as.numeric(logo2) +
    lang + as.numeric(IQv) + as.numeric(IQn)) * session, data = allmeans[allmeans$signal ==
    "speech", ])

Residuals:
     Min       1Q   Median       3Q      Max
-0.53109 -0.12613 -0.01707  0.13949  0.68430

Coefficients:
                                    Estimate Std. Error t value Pr(>|t|)
(Intercept)                       -0.6284977  1.6431547  -0.382   0.7030
age                                0.0223149  0.0106765   2.090   0.0396 *
CIthreshold                       -0.0088858  0.0175195  -0.507   0.6133
Ci1time                           -0.0114485  0.0119648  -0.957   0.3413
as.numeric(logo2)                  0.0535752  0.1323659   0.405   0.6867
lang                               0.0012244  0.0028715   0.426   0.6709
as.numeric(IQv)                    0.0052634  0.0094957   0.554   0.5808
as.numeric(IQn)                   -0.0004256  0.0085598  -0.050   0.9605
sessionleft ear                    0.2026919  2.2694245   0.089   0.9290
age:sessionleft ear               -0.0078980  0.0139520  -0.566   0.5728
CIthreshold:sessionleft ear        0.0191092  0.0254016   0.752   0.4539
Ci1time:sessionleft ear            0.0163948  0.0160473   1.022   0.3098
as.numeric(logo2):sessionleft ear -0.0766727  0.1807201  -0.424   0.6724
lang:sessionleft ear              -0.0003831  0.0040482  -0.095   0.9248
as.numeric(IQv):sessionleft ear   -0.0095191  0.0126289  -0.754   0.4531
as.numeric(IQn):sessionleft ear    0.0025264  0.0118482   0.213   0.8317
---
Signif. codes:  0 ‘***’ 0.001 ‘**’ 0.01 ‘*’ 0.05 ‘.’ 0.1 ‘ ’ 1

Residual standard error: 0.2259 on 86 degrees of freedom
  (10 observations deleted due to missingness)
Multiple R-squared:  0.3115,  Adjusted R-squared:  0.1914
F-statistic: 2.594 on 15 and 86 DF,  p-value: 0.002977
```

```
Anova Table (Type II tests)

Response: pupil
                   Sum Sq Df F value  Pr(>F)
age                0.3380  1  6.6245 0.01177 *
CIthreshold        0.0000  1  0.0003 0.98719
Ci1time            0.0044  1  0.0857 0.77039
as.numeric(logo2)  0.0010  1  0.0191 0.89050
lang               0.0133  1  0.2598 0.61157
as.numeric(IQv)    0.0000  1  0.0004 0.98498
as.numeric(IQn)    0.0012  1  0.0228 0.88041
session            0.1713  1  3.3576 0.07036 .
age:session        0.0163  1  0.3205 0.57281
CIthreshold:session 0.0289  1  0.5659 0.45393
```

```
Ci1time:session          0.0533  1  1.0438 0.30981
as.numeric(logo2):session 0.0092  1  0.1800 0.67243
lang:session             0.0005  1  0.0090 0.92482
as.numeric(IQv):session  0.0290  1  0.5681 0.45306
as.numeric(IQn):session  0.0023  1  0.0455 0.83165
Residuals                4.3875 86
---
Signif. codes:  0 '***' 0.001 '**' 0.01 '*' 0.05 '.' 0.1 ' ' 1
```

Model M3

```
Call:
lm(formula = pupil ~ (age + CIthreshold + Ci1time + as.numeric(logo2) +
    lang + as.numeric(IQv) + as.numeric(IQn)) * session, data = allmeans[allmeans$signal ==
    "music", ])

Residuals:
    Min      1Q  Median      3Q     Max
-0.5834 -0.1048  0.0033  0.1105  0.6202

Coefficients:
                                 Estimate Std. Error t value Pr(>|t|)
(Intercept)                     2.248e+00  1.712e+00   1.314   0.1925
age                            -5.357e-05  1.095e-02  -0.005   0.9961
CIthreshold                    -5.133e-03  1.911e-02  -0.269   0.7889
Ci1time                        -1.929e-02  1.256e-02  -1.535   0.1285
as.numeric(logo2)              -3.256e-01  1.355e-01  -2.403   0.0185 *
lang                           -3.354e-03  2.951e-03  -1.136   0.2590
as.numeric(IQv)                -1.111e-02  9.708e-03  -1.144   0.2558
as.numeric(IQn)                 4.456e-03  8.727e-03   0.511   0.6110
sessionleft ear                -1.120e+00  2.269e+00  -0.494   0.6229
age:sessionleft ear            -1.774e-03  1.393e-02  -0.127   0.8990
CIthreshold:sessionleft ear    -2.033e-02  2.635e-02  -0.772   0.4425
Ci1time:sessionleft ear         7.973e-03  1.639e-02   0.486   0.6279
as.numeric(logo2):sessionleft ear  4.400e-01  1.851e-01   2.378   0.0197 *
lang:sessionleft ear            2.499e-03  4.160e-03   0.601   0.5496
as.numeric(IQv):sessionleft ear  9.876e-04  1.335e-02   0.074   0.9412
as.numeric(IQn):sessionleft ear  4.304e-03  1.265e-02   0.340   0.7346
---
Signif. codes:  0 '***' 0.001 '**' 0.01 '*' 0.05 '.' 0.1 ' ' 1

Residual standard error: 0.2321 on 84 degrees of freedom
  (15 observations deleted due to missingness)
Multiple R-squared:  0.3129,  Adjusted R-squared:  0.1902
F-statistic:  2.55 on 15 and 84 DF,  p-value: 0.003577

Anova Table (Type II tests)

Response: pupil
                Sum Sq Df F value  Pr(>F)
age             0.0016  1  0.0290 0.86528
```

```
CIthreshold                 0.0779  1  1.4456 0.23262
Ci1time                     0.1766  1  3.2769 0.07384 .
as.numeric(logo2)           0.0509  1  0.9452 0.33374
lang                        0.0547  1  1.0155 0.31647
as.numeric(IQv)             0.1359  1  2.5219 0.11603
as.numeric(IQn)             0.0571  1  1.0590 0.30640
session                     0.0042  1  0.0787 0.77974
age:session                 0.0009  1  0.0162 0.89896
CIthreshold:session         0.0321  1  0.5953 0.44253
Ci1time:session             0.0128  1  0.2367 0.62790
as.numeric(logo2):session 0.3046  1  5.6535 0.01969 *
lang:session                0.0194  1  0.3610 0.54959
as.numeric(IQv):session   0.0003  1  0.0055 0.94122
as.numeric(IQn):session   0.0062  1  0.1157 0.73461
Residuals                   4.5258 84
---
Signif. codes:  0 ‘***’ 0.001 ‘**’ 0.01 ‘*’ 0.05 ‘.’ 0.1 ‘ ’ 1
```
